# Increasing Prevalence of Myopia in Europe and the Impact of Education

**DOI:** 10.1016/j.ophtha.2015.03.018

**Published:** 2015-07

**Authors:** Katie M. Williams, Geir Bertelsen, Phillippa Cumberland, Christian Wolfram, Virginie J.M. Verhoeven, Eleftherios Anastasopoulos, Gabriëlle H.S. Buitendijk, Audrey Cougnard-Grégoire, Catherine Creuzot-Garcher, Maja Gran Erke, Ruth Hogg, René Höhn, Pirro Hysi, Anthony P. Khawaja, Jean-François Korobelnik, Janina Ried, Johannes R. Vingerling, Alain Bron, Jean-François Dartigues, Astrid Fletcher, Albert Hofman, Robert W.A.M. Kuijpers, Robert N. Luben, Konrad Oxele, Fotis Topouzis, Therese von Hanno, Alireza Mirshahi, Paul J. Foster, Cornelia M. van Duijn, Norbert Pfeiffer, Cécile Delcourt, Caroline C.W. Klaver, Jugnoo Rahi, Christopher J. Hammond, European Eye Epidemiology (E) Consortium

**Affiliations:** 1 Department of Ophthalmology, King's College London, St. Thomas' Hospital, London, United Kingdom; 2 Department of Twin Research and Genetic Epidemiology, King's College London, St. Thomas' Hospital, London, United Kingdom; 3 Department of Ophthalmology, University Hospital of North Norway, Tromsø, Norway; 4 Department of Community Medicine, Faculty of Health Sciences, UiT The Arctic University of Norway, Tromsø, Norway; 5 Life Course, Epidemiology and Biostatistics Section, UCL Institute of Child Health, London, United Kingdom; 6 University Medical Center, Department of Ophthalmology, Mainz, Germany; 7 Department of Ophthalmology, Erasmus Medical Center, Rotterdam, The Netherlands; 8 Department of Epidemiology, Erasmus Medical Center, Rotterdam, The Netherlands; 9 Department of Ophthalmology, Aristotle University of Thessaloniki, Thessaloniki, Greece; 10 University Bordeaux, Bordeaux, France; 11 ISPED, Centre INSERM U897-Epidemiologie-Biostatistique, Bordeaux, France; 12 Department of Ophthalmology, Eye and Nutrition Research Group UMR 1324 INRA, University Hospital Dijon, France; 13 Department of Ophthalmology, Oslo University Hospital, Oslo, Norway; 14 Centre for Experimental Medicine, Institute of Clinical Science, Queen's University Belfast, Belfast, United Kingdom; 15 Department of Public Health and Primary Care, Institute of Public Health, University of Cambridge School of Clinical Medicine, Cambridge, United Kingdom; 16 Institute of Genetic Epidemiology, Helmholtz Center Munich, German Research Center for Environmental Health, Neuherberg, Germany; 17 London School of Hygiene and Tropical Medicine, London, United Kingdom; 18 Institute of Human Genetics, Klinikum rechts der Isar, Technische Universität, Munich, Germany; 19 Department of Ophthalmology, Nordland Hospital, Norway, Bodø, Norway; 20 NIHR Biomedical Research Centre, Moorfields Eye Hospital NHS Foundation Trust & UCL Institute of Ophthalmology, London, United Kingdom

**Keywords:** CI, confidence interval, D, diopters, E^3^, European Eye Epidemiology

## Abstract

**Purpose:**

To investigate whether myopia is becoming more common across Europe and explore whether increasing education levels, an important environmental risk factor for myopia, might explain any temporal trend.

**Design:**

Meta-analysis of population-based, cross-sectional studies from the European Eye Epidemiology (E^3^) Consortium.

**Participants:**

The E^3^ Consortium is a collaborative network of epidemiological studies of common eye diseases in adults across Europe. Refractive data were available for 61 946 participants from 15 population-based studies performed between 1990 and 2013; participants had a range of median ages from 44 to 78 years.

**Methods:**

Noncycloplegic refraction, year of birth, and highest educational level achieved were obtained for all participants. Myopia was defined as a mean spherical equivalent ≤−0.75 diopters. A random-effects meta-analysis of age-specific myopia prevalence was performed, with sequential analyses stratified by year of birth and highest level of educational attainment.

**Main Outcome Measures:**

Variation in age-specific myopia prevalence for differing years of birth and educational level.

**Results:**

There was a significant cohort effect for increasing myopia prevalence across more recent birth decades; age-standardized myopia prevalence increased from 17.8% (95% confidence interval [CI], 17.6–18.1) to 23.5% (95% CI, 23.2–23.7) in those born between 1910 and 1939 compared with 1940 and 1979 (*P* = 0.03). Education was significantly associated with myopia; for those completing primary, secondary, and higher education, the age-standardized prevalences were 25.4% (CI, 25.0–25.8), 29.1% (CI, 28.8–29.5), and 36.6% (CI, 36.1–37.2), respectively. Although more recent birth cohorts were more educated, this did not fully explain the cohort effect. Compared with the reference risk of participants born in the 1920s with only primary education, higher education or being born in the 1960s doubled the myopia prevalence ratio–2.43 (CI, 1.26–4.17) and 2.62 (CI, 1.31–5.00), respectively—whereas individuals born in the 1960s and completing higher education had approximately 4 times the reference risk: a prevalence ratio of 3.76 (CI, 2.21–6.57).

**Conclusions:**

Myopia is becoming more common in Europe; although education levels have increased and are associated with myopia, higher education seems to be an additive rather than explanatory factor. Increasing levels of myopia carry significant clinical and economic implications, with more people at risk of the sight-threatening complications associated with high myopia.

Myopia (near-sightedness) occurs when a distant object's image is formed anterior to the retinal plane, most commonly as a result of an increased axial length. This results in blurred distant vision and, unlike hyperopia, requires refractive correction at all ages and severity for clear focus. Myopia is already the most common eye condition worldwide, but the prevalence is significantly increasing, especially in Southeast Asia.^1–3^ In Europe, Australia, and the United States, the prevalence of myopia seems to be lower^4,5^; however, there is evidence of an increasing prevalence in the United States and elsewhere,^6–8^ particularly among young adults.^9^ This is of concern because myopia, even when appropriately corrected, is associated with an increased risk of sight-threatening diseases, such as myopic maculopathy, retinal detachment, glaucoma, and cataract.^10^ Myopic maculopathy is currently untreatable and already contributes to visual impairment in working-age adults.^11^ Increasing myopia levels in Europe carry implications for public health policy in both the provision of clinical services and the economic sequelae from the resulting visual impairment among the working population.

Myopia is a highly heritable trait,^12,13^ and to date a number of genetic polymorphisms have been associated with refractive error, albeit explaining only a small proportion of this heritability.^14,15^ Environmental factors play a key role in myopia development and must explain the recent changes in prevalence.^16^ Myopia has been associated with education, near work, urbanization, prenatal factors, socioeconomic status, cognitive ability, season of birth, light, and time spent outdoors.^2,16–25^ One of the strongest and most replicated risk factors is educational attainment,^16,26^ and there is some evidence of interaction between genetic factors and education influencing the risk of myopia.^27^ The increased levels of higher education over the 20th century^28^ might be a causative factor, or marker of a causative factor, for increasing myopia prevalence.

The aims of this study are to identify whether myopia is becoming more common across Europe and to examine whether increasing levels of education explain any temporal trend, using data from more than 60 000 participants from the European Eye Epidemiology (E^3^) Consortium.

## Methods

### Study Population

The E^3^ consortium is a collaborative initiative to share and meta-analyze epidemiologic data on common eye diseases across Europe. Thirty-three studies are currently part of the consortium, and a range of ophthalmic data are available on approximately 124 000 individuals from population-based and case-control cohorts. All studies adhere to the tenets of the Declaration of Helsinki, and relevant local ethical committee approvals with specific study consent were obtained.

Refractive error measurements from 68 350 adults within the 15 E^3^ population-based studies that had data on refractive error were included. These included population-based cross-sectional or cohort studies, with 2 studies recruiting participants nationally and 13 studies recruiting from a local population. Further details on each study are provided in Table 1 and the Supplementary information (available at www.aaojournal.org). Exclusion criteria included subjects who had cataract or refractive surgery, retinal detachment, or other conditions, such as keratoconus, which might influence refraction (n = 6404). Data on age at refraction and birth year were available for 61 946 individuals, with information on education level for 60 125 subjects. Participants were mainly middle to late age; 98% were of European descent (where ethnicity was known), predominantly from Northern and Western Europe; and refractive examinations were performed from 1990 to 2013 (Table 1).

### Study Variables

Noncycloplegic refractions were performed on all individuals using subjective refraction, autorefraction, or a combination of focimetry with subjective refraction. Spherical equivalent was calculated using the standard formula (spherical equivalent = sphere + [cylinder/2]). Myopia was defined as ≤−0.75 diopters. Myopia prevalence by age was calculated, using 5- and 10-year age bands from ≥15 years to ≥90 years. To study the impact of education on myopia, given the variation in educational systems across Europe, we established a simplified 3-tier level of education across all cohorts. Primary education was defined as those leaving school before 16 years of age, secondary education was defined as those leaving education up to the age of 19 years, and higher education was defined as those leaving education at or after the age of 20 years. Those aged younger than 20 years at the time of refraction (and therefore unable to have reached the highest education tier) were excluded from this analysis to avoid misclassification bias.

We investigated the evidence for a cohort effect on increasing myopia prevalence by observing variations in myopia prevalence within defined age bands. These analyses are focused on the age range constituting the majority of our cohort (40–80 years of age, birth year 1910–1979, n = 56 088), meaning the youngest and oldest participants, for whom we had no comparative birth cohort, were not considered. Prevalence between different birth cohorts was examined, initially using decade bins (1910–1970) and subsequently in 2 birth cohort groups divided by the median birth decade (1940–1949). Finally we examined the influence of education by examining the myopia prevalence between birth cohorts with the additional stratification of educational status.

### Statistical Analysis

Study-specific summary data for myopia prevalence were obtained and combined in a random-effect meta-analysis stratified by age. A random-effects model was chosen over a fixed-effects model to allow for expected heterogeneity between studies as a result of varying study design. Age was standardized with demographic distribution adjustments to age-specific estimates according to the European Standard Population 2010.^29^ Evidence for the presence of a cohort effect was investigated using random-effect meta-analyses of myopia prevalence stratified by age and birth year, and subsequently age, birth year, and educational level. Differences between estimates of myopia prevalence were evaluated using the analysis of variance test, proportion *z* tests, and prevalence ratios (relative difference in prevalence against a defined baseline). Differences were considered significant at *P* < 0.05.

Statistical analysis was performed using Stata statistical software version 13.1 (StataCorp LP, College Station, TX). Graphical outputs^30^ were obtained using Stata, Origin version 9.0 (OriginLab Corp, Northampton, MA), or ggplot2(30) in R software (R Foundation for Statistical Computing, Vienna, Austria; available at http://www.R-project.org).

## Results

In this meta-analysis of 61 946 adults, the overall myopia prevalence was 24.3% (95% confidence interval [CI], 20.1–28.5), with an age-standardized prevalence in Europe of 30.6% (95% CI, 30.3–30.8). Age-stratified analyses^31^ revealed a high prevalence in young adults (47.2% [95% CI, 41.8–52.5] in those aged 25–29 years), which was almost double the prevalence in those of middle to older age (27.5% [95% CI, 23.5–31.5] in those aged 55–59 years). There were no significant differences in the myopia prevalence by gender.^31^

### Cohort Effect for Increasing Myopia Prevalence

**Figure 1:**
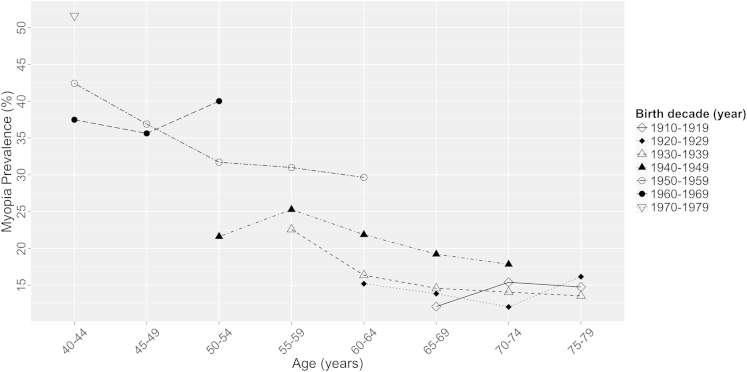
Prevalence of myopia (spherical equivalent ≤−0.75 diopters) against age stratified by decade of birth. Individuals aged 40 to 79 years included.

There was a trend of higher myopia prevalence with more recent birth decade across all age groups (Fig 1), although sample sizes for some point estimates were low, resulting in wide CIs (Table 2, available at www.aaojournal.org).

**Figure 2:**
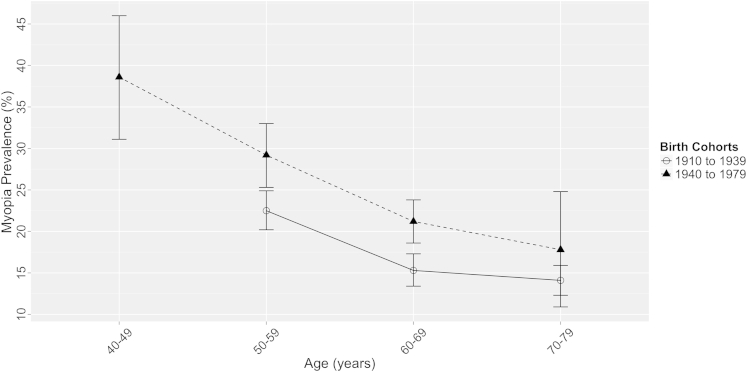
Prevalence of myopia (spherical equivalent ≤−0.75 diopters) as a function of age for 2 birth cohorts (1910–1939, 1940–1979) with 95% confidence intervals.

We examined the prevalence of myopia in 2 birth cohort groups (divided by the median birth decade): those born between 1910 and 1939 (n = 22 660) and those born between 1940 and 1989 (n = 33 428) (Fig 2). Myopia prevalence in a variance model was significantly higher in the more recent birth cohort group (*P* = 0.03). Age-standardized myopia prevalence over a comparable age range of 50 to 79 years increased from 17.8% (95% CI, 17.6–18.1) in those born in 1910–1939 to 23.5% (95% CI, 23.2–23.7) in those born in 1940–1979. In age-specific analyses, the prevalence of myopia in those aged 50 to 59 years (at the time of their refraction) was 22.5% (95% CI, 20.2–24.9) in those born before 1940, compared with 29.2% (95% CI, 25.3–33.0) in those born after 1940 (*P* = 0.004). A similar significant increase of 15.3% (95% CI, 13.4–17.3) to 21.2% (95% CI, 18.6–23.8) was observed in those aged 60 to 69 years (*P* < 0.001).

### Influence of Education on Myopia Risk and the Cohort Effect

**Figure 3:**
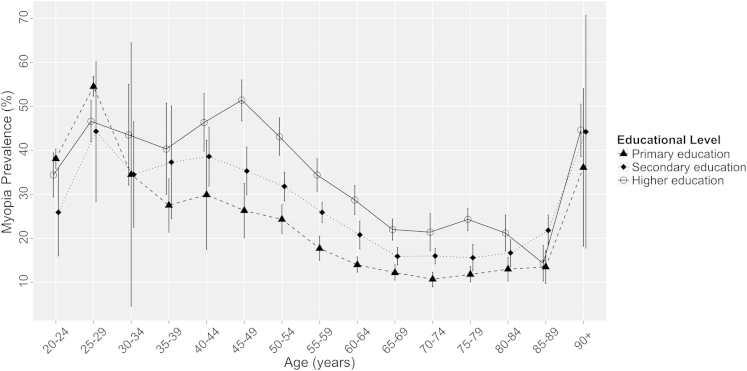
Prevalence of myopia (spherical equivalent ≤−0.75 diopters) with 95% confidence interval stratified by highest educational level achieved: primary education, leaving education at age <16 years; secondary education, leaving school at age ≤19 years; higher education, leaving school at age ≥20 years.

The association between education and myopia was investigated in the 13 studies from which these data were available (n = 60 125 participants). Educational level was significantly associated with myopia prevalence across all age strata (*P* < 0.0001). Overall, the age-standardized myopia prevalence for those completing primary, secondary, and higher education was 25.4% (95% CI, 25.0–25.8), 29.1% (95% CI, 28.8–29.5), and 36.6% (95% CI, 36.1–37.2), respectively. In those aged 35 to 84 years, the majority of study subjects, myopia prevalence in participants with higher education was approximately double those with primary education (Fig 3). For example, in subjects aged 45 to 49 years when tested, the myopia prevalence was 26.3% (95% CI, 20.1–32.5) compared with 51.4% (95% CI, 46.7–56.0) for those with primary and higher education, respectively, and in those aged 60 to 64 years, myopia prevalence was 14.0% (95% CI, 12.3–15.8) compared with 28.7% (95% CI, 25.4–32.0) for those with primary and higher education, respectively. The trends observed are less clear in younger subjects (<35 years) in Figure 3, most likely because of small sample sizes (n = 216 aged 20–25 years, n = 336 aged 25–30 years), which are further stratified by education level with corresponding wide CIs.

**Figure 4:**
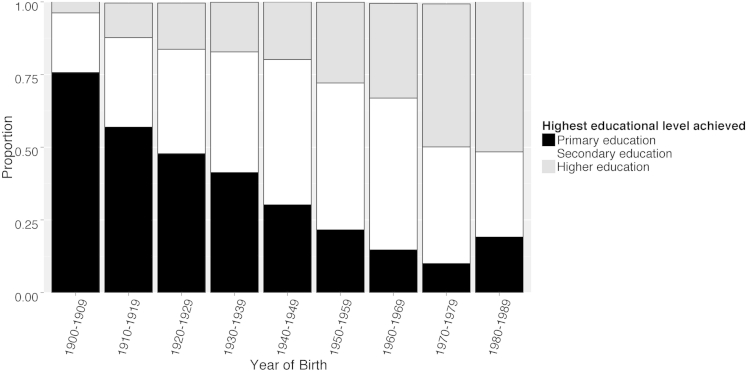
Distribution of highest educational level achieved, stratified by year of birth (1900–1989): primary education, leaving education at age <16 years; secondary education, leaving school at age ≤19 years; higher education, leaving school at age ≥20 years.

Levels of education throughout Europe have increased in the past 90 years (Fig 4). The proportion of individuals progressing to higher education increased from 4% of those born in the 1900s to 16% in the 1920s, 20% in the 1940s, 33% in the 1960s, and approximately 61% in the 1980s.

**Figure 5:**
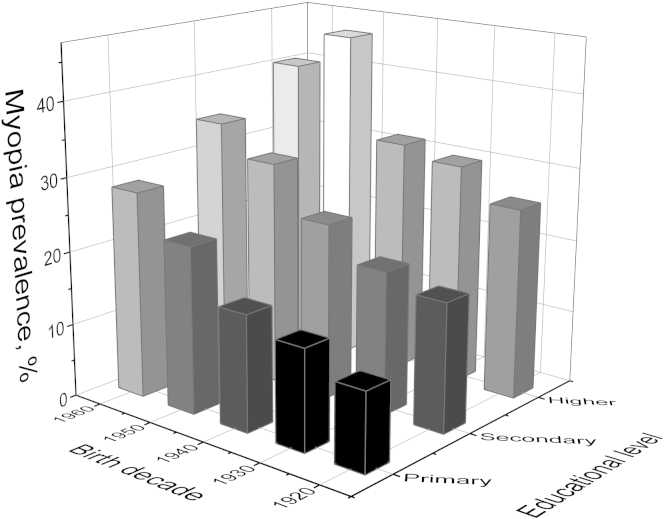
Myopia prevalence (spherical equivalent ≤−0.75 diopters) by birth cohort and educational level in individuals aged 45 to 65 years: primary education, leaving education at age <16 years; secondary education, leaving school at age ≤19 years; higher education, leaving school at age ≥20 years.

However, although those born more recently were more likely to have achieved a higher educational level, this alone did not explain the cohort effect of increasing myopia. As shown in Figure 5, for individuals aged 45 to 65 years (age range selected for minimal age-related myopia variance and large available sample size), the increase in myopia prevalence with a more recent birth decade was observed across all educational groups. This was most pronounced for participants achieving only a primary education, in whom myopia prevalence increased from 10.7% (95% CI, 7.6–13.8) to 28.1% (95% CI, 18.1–38.0) between birth decades 1920 to 1929 and 1960 to 1969 (*P* = 0.001). The corresponding increase in myopia in those with higher education was from 26.0% (95% CI, 17.4–34.6) to 40.2% (95% CI, 30.5–50.0) (*P* = 0.03). Compared with the reference risk of participants with primary education and born in the 1920s, the myopia prevalence ratio for those achieving a higher education was 2.43 (95% CI, 1.26–4.17) and for those born in the 1960s was 2.62 (95% CI, 1.31–5.00). Individuals born in the 1960s and completing higher education had approximately 4 times the baseline risk, with a prevalence ratio of 3.76 (95% CI, 2.21–6.57). Thus, the individual associations of educational level and birth cohort had an additive effect on myopia prevalence.

## Discussion

Our study provides the first evidence that myopia is becoming more common across Western and Northern Europe, with a clear trend of higher myopia prevalence in participants with a more recent birth year (Fig 1). This is similar to the increase reported in North America and, albeit to a lesser extent, Southeast Asian populations.^6,7,32,33^ Evidence of increasing myopia prevalence carries clinical and economic implications. The increased requirement for detection and treatment of myopia, entailing glasses, contact lenses, or more recently laser refractive surgery, has significant implications for clinical optometric and ophthalmic service provision, and the health care system. Additional ophthalmic services will be needed for treatable sight-threatening complications, such as retinal detachment, glaucoma, and cataract.^10,34^ The increasing prevalence of myopia also implies that untreatable complications, such as myopic maculopathy, most commonly seen in high myopia, will become more common. This will result in more visual impairment in middle- to older-aged individuals, including a proportion of the working-age population, with consequent economic implications.

Myopia has been strongly associated with education,^2,21,24,35^ and we explored this using a simple 3-tier classification of educational level. Increasing educational level had a strong effect, with myopia twice as common in those achieving a higher education compared with participants leaving school before 16 years of age. There was a clear trend of increasing prevalence of myopia across the tiers of education level, suggesting a potential additive effect of years of education. This interesting association may reflect a number of factors: greater near work activities with more education and less time in outdoor light, shared genetic factors underlying myopia and intelligence, or factors related to educational opportunity, such as socioeconomic status or maternal nutrition. These associations have been explored in younger cohorts,^18–21,36,37^ although causal pathways are yet to be fully understood.

Reasons for the observed cohort effect are clearly multifactorial, and education is an obvious possible explanation; in our data, only 12% of participants born in the 1920s went on to higher education, compared with 33% born in the 1960s. This educational expansion has been observed across Europe in both men and women, with a sharp trajectory toward mass higher education after World War II.^28,38^ In addition to the disruption of education and economic consequences of World War II, adverse health outcomes have been reported in young people growing up at that time, notably diabetes, depression, and heart disease.^39^ Although there is no known direct link between these health issues and myopia, the deprivation may have affected eye growth and resulting refraction. Certainly there was an increase in myopia in subjects born after 1950, but it is difficult to be certain what aspect of the seismic changes in Europe after the war might be responsible.

Although the younger generations were more educated, we found a clear increase in the prevalence of myopia across the birth cohorts within each educational stratum, as well as the additive effect of educational status. Therefore, increasing levels of myopia were not explained by education alone, and a more recent birth year and higher educational level had an additive effect on myopia risk. Our simple 3-tier education stratification may be subject to residual confounding from variation in educational practices, and it may be these, rather than changes in education level, that are contributing to the observed cohort effect. In the latter half of the last century, there was increasing use of computers, increasing length of the educational day with increased after-school tuition, and less outdoor play as a result of reduced recess time.^35^

### Study Limitations

The E^3^ consortium has provided a large data set to meta-analyses’ temporal trends and educational associations for myopia prevalence across Europe. Limitations to this consortium meta-analysis include heterogeneity between studies. Contributing studies inherently differed in study design and cohort sampling. In acknowledgment of this heterogeneity, we performed a random-effect rather than a fixed-effect meta-analysis, assuming no fixed effect between studies. There are also differences between European countries in terms of urbanization, economy, social class, education, and lifestyle, which are known to influence myopia. Data on these variables at an individual or study-specific level were not uniformly available, and data often were collected from middle-aged and older participants, so retrospective collection of potential contributing factors such as outdoor exposure, amount of reading, and area of residence during the critical first 20 years of refractive error development would be impossible. In addition, potential multicollinearity of these likely highly correlated factors (e.g., reading and education) would make assessment of separate effects difficult. In an attempt to reduce heterogeneity arising from these associated factors, we stratified the random-effects meta-analysis by age and educational level (both significantly associated with myopia). Applicability of our findings is greatest for middle- to older-aged individuals and for those from Northern and Western European countries, given the sampled ages and the location of the E^3^ studies (Table 1), although ultimately the degree to which these studies are representative of the underlying population is unknown.

Further limitations include the crude nature in which education was classed, which as previously acknowledged may result in residual confounding. In addition, education status was collected retrospectively and therefore prone to recall error, possibly heightened in older participants. Refractions were all noncycloplegic, although this is reasonable given the age of participants.^40,41^ Finally, these data are not longitudinal, so we have not examined reasons for the lower prevalence with age within birth decades, although the cohort effect we identified may be part of this explanation. Other reasons include the well-known hyperopic shift with age and could include other factors, such as censoring with age if myopic subjects receive earlier cataract surgery.

In conclusion, the prevalence of myopia is increasing in Europe, a finding that is not fully explained by increasing education levels despite higher educational achievement being associated with myopia and becoming more widespread in Europe. The changes in prevalence are similar to those observed in North America, although they remain far less than those identified in Southeast Asia, possibly because of differing intensity of education from an early age.^1,6,35^ High levels of myopia were detected in the younger adults with a more recent birth year, of whom approximately half were affected. This has significant implications for the future; increasing myopia prevalence, and specifically high levels in younger individuals, will potentially result in an increasing burden of associated visual impairment in the future.

## Figures and Tables

**Table 1:** Description of the 15 European Eye Epidemiology Consortium Studies Included in this Meta-Analysis of Refractive Error

Study	Data Collection Period	Study Design	Total Participants with Refraction	Refraction Method	Exclusions∗ (Cataract Surgery)	Total Participants Included	Median Age, yrs (Range)	Gender, % Female	Ethnicity, % European (% Unknown)	Higher Education, %	Crude Myopia Prevalence, %
Northern Europe											
1958 British Birth Cohort, UK	2002–2003	Population-based birth cohort (N)	2502	Autorefraction	7 (0)	2495	44 (44–46)	51.7	98.0 (9.2)	29.9	48.7
EPIC-Norfolk, UK	2004–2011	Population-based cross-sectional study (L)	8508	Autorefraction	1110 (971)	7444	67 (48–92)	54.5	99.7 (0)	17.9	23.0
Tromsø Eye Study, Norway	2007–2008	Population-based cohort (L)	6565	Autorefraction	773 (700)	5792	61 (38–87)	55.9	NA (100)	32.5	19.4
TwinsUK, UK	1998–2010	National twin cohort (N)	6245	Autorefraction	161 (61)	6095	55 (16–85)	91.2	98.2 (23.9)	22.3	31.4
Southern Europe											
Thessaloniki Eye Study, Greece	1999–2005	Cross-sectional population-based study (L)	2259	Subjective	316 (303)	1952	69 (60–94)	44.7	100 (0)	Unknown	14.2
Western Europe											
ALIENOR, France	2006–2008	Population-based cohort (L)	951	Autorefraction	333 (318)	618	79 (73–93)	56.6	NA (100)	20.0	16.7
ERF, Netherlands	2002–2005	Family-based cross-sectional study (L)	2708	Subjective	46 (45)	2662	49 (14–87)	55.1	100 (0)	16.9	21.2
Gutenberg Health Study, Germany	2007–2012	Population-based cohort (L)	14 679	Autorefraction	610 (610)	14 069	54 (35–74)	49.4	NA (100)	37.6	31.9
KORA, Germany	2004–2005	Population-based cohort (L)	3078	Autorefraction	706 (177)	2372	55 (35–84)	50.4	100 (0)	14.7	36.1
Montrachet, France	2009–2013	Population-based cohort (L)	1143	Autorefraction	584 (562)	576	81 (76–92)	57.5	NA (100)	Unknown	19.1
Rotterdam Study I, Netherlands	1990–1993	Population-based cohort (L)	6748	Subjective	182 (172)	6566	68 (55–106)	59.3	98.5 (2.0)	11.6	16.4
Rotterdam Study II, Netherlands	2000–2002	Population-based cohort (L)	2689	Subjective	110 (110)	2579	62 (55–99)	54.8	87.8 (0.1)	22.3	21.9
Rotterdam Study III, Netherlands	2005–2008	Population-based cohort (L)	3624	Subjective	94 (74)	3530	56 (46–97)	56.3	NA (100)	31.4	32.5
POLA, France	1995–1997	Population-based cohort (L)	2464	Autorefraction	157 (128)	2315	70 (60–93)	55.8	NA (100)	7.3	16.2
Mixed											
EUREYE: Norway, UK, France, Italy, Greece, and Estonia	2000–2002	Population-based cross-sectional survey in 7 cities (L)	4187	Autorefraction or focimetry with subjective refraction	1305 (517)	2882	72 (65–95)	56.7	NA (100)	30.0	15.6
Total cohort	1990–2013		68 350		6404 (4748)	61 946	62	57.6	98.1	36.0	25.8

ALIENOR = Antioxydants, Lipides Essentiels, Nutrition et maladies OculaiRes Study; EPIC = European Prospective Investigation into Cancer; ERF = Erasmus Rucphen Family Study; EUREYE = European Eye Study; KORA = Kooperative Gesundheitsforschung in der Region Augsburg; L = local; N = national; NA = not available; POLA = Pathologies Oculaires Liées à l'Age Study. Myopia classified in those with refraction ≤−0.75 diopters. ∗Exclusions = cataract surgery, refractive surgery, retinal detachment or other conditions affecting refraction.

## Supplementary Data

Supplementary Information

Author Study Group
