# Supplementary material for: Increasing Prevalence of Myopia in Europe and the Impact of Education
Source: Ophthalmology. 2015 Jul;122(7):1489–97. doi: 10.1016/j.ophtha.2015.03.018 (PMC4504030; doi:10.1016/j.ophtha.2015.03.018)
Supplement: Table 2 [file mmc1.pdf]

	Birth decade						
**Age**	**1910-1919**	**1920-1929**	**1930-1939**	**1940-1949**	**1950-1959**	**1960-1969**	**1970-1979**
40-44					42.4 (21.6-63.2)	37.5 (25.7-49.3)	51.6 (22.4-80.9)
45-49				*	36.9 (27.6-46.2)	35.6 (28.5-42.8)	
50-54				21.6 (14.2-29.1)	31.7 (27.0-36.5)	40.0 (33.0-47.0)	
55-59			22.6 (20.2-25.0)	25.3 (21.9-28.7)	31.0 (28.1-33.9)		
60-64		15.2 (12.7-17.7)	16.4 (13.2-19.5)	21.9 (18.8-25.0)	29.7 (18.9-40.4)		
65-69	12.1 (9.5-14.7)	13.9 (11.2-16.5)	14.6 (12.0-17.1)	19.2 (16.5-21.9)			
70-74	15.4 (13.0-17.8)	12.1 (8.8-15.3)	14.1 (11.4-16.7)	17.8 (10.9-24.8)			
75-79	14.8 (10.9-18.6)	16.2 (13.6-18.7)	13.6 (10.4-16.7)				

Table 2 Prevalence of myopia (Spherical Equivalent ≤ -0.75 Diopters) against birth year stratified by age.
Individuals aged 40 to 79 included. (* = meta-analysis not possible due to single contributing prevalence estimate)
