# Supplementary material for: Increasing Prevalence of Myopia in Europe and the Impact of Education
Source: Ophthalmology. 2015 Jul;122(7):1489–97. doi: 10.1016/j.ophtha.2015.03.018 (PMC4504030; doi:10.1016/j.ophtha.2015.03.018)
Supplement: Supplementary Information [file mmc2.pdf]

Supplementary information

The Alienor study

The Alienor (Antioxydants, Lipides Essentiels, Nutrition et maladies OculaiRes) Study is a population-based prospective study aiming at assessing the associations of age-related eye diseases (age-related maculopathy, glaucoma, cataract, dry eye syndrome) with nutritional factors (in particular antioxidants, macular pigment and fatty acids), determined from plasma measurements and estimation of dietary intakes. It also takes into account other major determinants of eye diseases, including gene polymorphisms, environmental factors and vascular factors. The methods of this study have been published elsewhere (1).

Subjects of the Alienor Study were recruited from an on-going population-based study on the vascular risk factors for dementia, the Three-City (3C) Study (2). The 3C Study included 9,294 subjects aged 65 years or more from three French Cities (Bordeaux, Dijon and Montpellier), among whom 2,104 were recruited in Bordeaux. They were initially recruited in 1999-2001 and followed-up about every two years since. The Alienor Study consists of eye examinations, which are proposed to all participants of the 3C cohort in Bordeaux since the third follow-up (2006-2008). Among the 1,450 participants re-examined between October 2006 and May 2008, 963 (66.4%) participated in the Alienor Study's baseline eye examination. The design of this study has been approved by the Ethical Committee of Bordeaux (Comité de Protection des Personnes Sud-Ouest et Outre-Mer III) in May 2006.

1958 British Birth Cohort

The 1958 British Birth Cohort originally consisted of 17,000 individuals, all born in one week in March 1958, and subsequently followed up at intervals with physical examination and collection of data on environmental, social and lifestyle factors (3). In 2002/3, when the participants were aged 44/45 years, a biomedical survey was undertaken of the 9377 individuals still actively participating in the study. Of all the subjects, 2502 were selected randomly and underwent non-cycloplegic autorefraction, using the Retinomax 2 autorefractor (equipment costs precluded autorefraction of all subjects).

EPIC-Norfolk Eye Study

The European Prospective Investigation into Cancer (EPIC) study is a pan-European prospective cohort study designed to investigate the aetiology of major chronic diseases (4) EPIC-Norfolk , one of the UK arms of EPIC, recruited and examined 25,639 participants aged 40-79 years between 1993 and 1997 for the baseline examination (5). Recruitment was via general practices in the city of Norwich and the surrounding small towns and rural areas, and methods have been described in detail previously (6). Since virtually all residents in the UK are registered with a general practitioner through the National Health Service, general practice lists serve as population registers. Ophthalmic assessment formed part of the third health examination and this has been termed the EPIC-Norfolk Eye Study (7). In total, 8,623 participants were seen for the ophthalmic examination, between 2004 and 2011. Refractive error was measured using an autorefractor (Model 500, Humphrey Instruments, San Leandro, California, USA. The EPIC-Norfolk Eye Study was carried out following the

principles of the Declaration of Helsinki and the Research Governance Framework for Health and Social Care. The study was approved by the Norfolk Local Research Ethics Committee (05/Q0101/191) and East Norfolk & Waveney NHS Research Governance Committee (2005EC07L).

Erasmus Rucphen Family (ERF) Study

The ERF Study is a family-based cohort in a genetically isolated population in the southwest of the Netherlands with over 3,000 participants aged between 18 and 86 years. The rationale and study design of this study have been described elsewhere (8, 9). Cross-sectional examination took place between 2002 and 2005. Refractive error was measured using a Topcon RM-A2000 autorefractor (non-dilated). All measurements in these studies were conducted after the Medical Ethics Committee of the Erasmus University had approved the study protocols.

EUREYE Study

The EUREYE Study is multicentre, population-based cross-sectional study with retrospective and current exposure measurements primarily designed to study age-related macular degeneration. A detailed description of the study design has been reported (10). Briefly, 7 study centres, Bergen, Norway; Tallinn, Estonia; Belfast, Northern Ireland, United Kingdom; Paris-Creteil, France; Verona, Italy; Thessaloniki, Greece; and Alicante, Spain, were chosen primarily to maximize the range of latitude and lifestyle behaviours, including diet. Refractive data was not collected from the Spanish centre. The EUREYE Study aimed to enrol 800 to 900 persons 65 years and older in each of the 7 centres. The sample size calculations estimated that 6000 people would be required to detect a prevalence of AMD of mean ± SD 2% ± 0.5% at 95% confidence and a design effect of 2, to allow for the cluster (ie, country) effects. The sampling frame consisted of all persons 65 years or older who were included in the National Population Registry (Estonia), Patient Register (Northern Ireland, which includes all people registered with family physicians—around 98% of the local population), National Office for Statistics (Spain), and Municipal Register (France, Greece, Italy, and Norway) at the time the sample was requested. In each centre, the random sample was drawn by the statistical officers at the registries. Ethics approval was obtained at each centre from the relevant ethics committee. Study participants gave informed written consent prior to participation. Any participant who was unable to achieve a 0.3 logMAR (Snellen 20/40) in either eye underwent automatic or manual retinoscopy followed by refraction and recording of best-corrected acuity.

Gutenberg Health Study

The Gutenberg Health Study (GHS) is an on-going, population-based, prospective, observational cohort study in the Rhine-Main Region in Midwestern Germany with a total of 15,010 participants (11). The study sample was recruited from subjects aged between 35 and 74 years at the time of the examination. Exclusion criteria were insufficient knowledge of the German language to understand explanations and instructions, and physical or psychic inability to participate in the examinations in the study centre. Refractive error was measured non-dilated using a Humphrey® Automated Refractor/Keratometer (HARK) 599 (Carl Zeiss Meditec, Jena, Germany). The study was approved by the Medical Ethics Committee of the University Medical Center Mainz and by the local and federal data safety commissioners.

KORA Study

KORA ("Kooperative Gesundheitsforschung in der Region Augsburg" which translates as "Cooperative Health Research in the Region of Augsburg") is a population-based study of adults randomly selected from 430,000 inhabitants living in Augsburg and 16 surrounding counties in Germany (12). The collection was done in 4 separate groups from 1984-2001 (S1-S4). All survey participants are residents of German nationality identified through the registration office. In the KORA S3 and S4 studies 4,856 and 4,261 subjects have been examined implying response rates of 75% and 67%, respectively. 3,006 subjects participated in a 10-year follow-up examination of S3 in 2004/05 (KORA F3), and 3080 of S4 in 2006/2008 (KORA F4). The age range of the participants was 25 to 74 years at recruitment. The study was approved by the local ethics committee. Written informed consent was obtained from all participants before enrolment in accordance with the Declaration of Helsinki. For each subject, eyeglass prescriptions were measured in addition to an evaluation using the Nikon Retinomax. Refractive error was analysed, taking the mean measured spherical equivalent (SE) across both eyes (or SE in a single eye when both eyes were not measured) as the trait of interest.

The Montrachet 3C Study

Subjects of the Montrachet (Maculopathy Optic Nerve nuTRition neurovAsCular and HEarT diseases) study were recruited from an on-going population-based study, the Three-City (3C) study, on the vascular risk factors for dementia (2). The 3C-Study was designed to examine the relationship between vascular diseases and dementia in a community housing 9,294 persons aged 65 years and over. The participants were selected from the electoral rolls and were only urban since they lived in 3 French cities, Bordeaux, Dijon and Montpellier. The 3C-Study began in 1999 and participants were evaluated every two years. A subgroup underwent ocular assessment in Bordeaux (Alienor study; see above (1)) and Dijon (Montrachet study).

In Dijon 4,934 subjects participated to the first run of the 3C-Study in 1999. They were followed every 2 years and at the fourth run undertaken in 2006/2007 they were still 3,137. Among them, 1,604 (51.1%) underwent an MRI at baseline and at the fourth year. We decided to include preferentially the participants having had an MRI and to complete the recruitment with participants without MRI. Therefore from October 22th, 2009 until March 31th, 2013, 913 volunteers with an MRI were recruited in the Montrachet study and 236 without and MRI. After approval by the regional ethics committee, the study was registered as 2009-A00448-49. Refractive error was determined using an autorefractor without cycloplegia (Tonoref II, Nidek, Aichi, Japan).

The POLA Study

The Pathologies Oculaires Liées à l'Age (POLA) Study is a population-based study aimed at identifying the risk factors of age-related eye diseases. The methods of this study have been published elsewhere (13). For inclusion in the study, participants needed to be a resident of Sète (South of France) and aged 60 years and over. According to the 1990 population census, there were almost 12,000 eligible residents, of whom our objective was to recruit 3,000. The population was informed of the study through the local media. We also contacted 4,543 residents individually by mail and telephone, using the electoral roll. The baseline examinations took place in a mobile unit equipped

with ophthalmologic devices. Between June 1995 and July 1997, 2,584 participants were recruited. The study was approved by the ethics committee of the University Hospital of Montpellier, France.

Refractive error was measure using a Topcon RM-A7000 autorefractor, and refined subjectively when assessing best-corrected visual acuity. Cataract surgery was ascertained by the absence of the natural lens at slit lamp.

The Rotterdam Study I/II/III

The Rotterdam Study is a population-based study established in Rotterdam, the Netherlands (14). It consists of three cohorts. The original cohort, RS-I, started in 1990 and includes 7,983 subjects aged 55 years and older. The second cohort, RS-II, was added in 2000 and includes 3,011 subjects aged 55 years and older. The last cohort, RS-III, includes 3,932 subjects of 45 years of age and older and started in 2006. Refractive error was measured using a Topcon RM-A2000 autorefractor (non-dilated). Other ophthalmic baseline and follow-up examinations, which are still on-going, were described previously (15). The Rotterdam Study has been approved by the institutional review board (Medical Ethics Committee) of the Erasmus Medical Center and by the review board of The Netherlands Ministry of Health, Welfare and Sports.

Thessaloniki Eye Study

The Thessaloniki Eye Study (TES) is a cross-sectional, population-based, epidemiologic study of chronic eye diseases in the Greek population of Thessaloniki. According to the National Statistical Service of Greece, Thessaloniki which is a major urban centre in Northern Greece is considered representative of the general population in the country. The initial recruitment frame of the TES consisted of 5,000 people, 60 years of age or older, who were identified randomly in February 1999 from approximately 321,000 persons registered in the municipality registers of the city of Thessaloniki. Subject recruitment is described in detail elsewhere (16). In summary, randomization was provided by the municipality statistical service. From the initial recruitment sample of the 5,000 names, 3,617 subjects were eligible and finally 2,554 participated in the study (participation rate 71%) (17). Study examination and data collection ended in March 2005. The study was approved by the Aristotle University Hospital Ethics Committee and the University of California Los Angeles Human Subject Protection Committee. If visual acuity was less than 20/30 with habitual correction, a full refraction was performed, and best-corrected visual acuity was measured.

Tromsø Eye Study

The Tromsø Eye Study (2007-08) is a substudy of the sixth Tromsø Study, a large comprehensive longitudinal population-based study started in 1974. The Tromsø Study and the cohort profile have been described elsewhere (18). The study sample was based upon the official population registry and includes a combination of whole birth cohorts and random selections among residents of the municipality of Tromsø. Refraction was measured by Nidek AR 660A autorefractor (Nidek Co., Ltd. Gamagori, Japan). Spherical equivalent was calculated as spherical power plus half the cylindrical power in dioptres (D) and presented as the mean value of left and right eye. The study design and methodology have been described in detail elsewhere (19).

TwinsUK

The TwinsUK adult twin registry, based at St. Thomas' Hospital in London, compromises over 12,000 predominantly female Caucasian ancestry twins, from throughout the United Kingdom (20). Twins largely volunteered unaware of the eye studies at the time of enrolment and gave fully informed consent under a protocol reviewed by the St. Thomas' Hospital Local Research Ethics Committee (EC04/015), which was performed in accordance with the Helsinki Declaration. Various eye phenotypes have been collected on a subset of twins. Refractive error was measured using non-cycloplegic autorefraction (ARM-10 autorefractor, Takagi Seiko, Japan).

References

1. Delcourt C, Korobelnik JF, Barberger-Gateau P, Delyfer MN, Rougier MB, Le Goff M, et al. Nutrition and age-related eye diseases: the Alienor (Antioxydants, Lipides Essentiels, Nutrition et maladies OculaiRes) Study. The journal of nutrition, health & aging. 2010;14(10):854-61. PubMed PMID: 21125205; PubMed Central PMCID: PMC3081304.
2. Group CS. Vascular factors and risk of dementia: design of the Three-City Study and baseline characteristics of the study population. Neuroepidemiology. 2003;22(6):316-25. PubMed PMID: 14598854.
3. Power C, Elliott J. Cohort profile: 1958 British birth cohort (National Child Development Study). International journal of epidemiology. 2006;35(1):34-41. doi: 10.1093/ije/dyi183. PubMed PMID: 16155052.
4. Riboli E, Kaaks R. The EPIC Project: rationale and study design. European Prospective Investigation into Cancer and Nutrition. International journal of epidemiology. 1997;26 Suppl 1:S6-14. PubMed PMID: 9126529.
5. Day N, Oakes S, Luben R, Khaw KT, Bingham S, Welch A, et al. EPIC-Norfolk: study design and characteristics of the cohort. European Prospective Investigation of Cancer. British journal of cancer. 1999;80 Suppl 1:95-103. PubMed PMID: 10466767.
6. Hayat SA, Luben R, Keevil VL, Moore S, Dalzell N, Bhaniani A, et al. Cohort profile: A prospective cohort study of objective physical and cognitive capability and visual health in an ageing population of men and women in Norfolk (EPIC-Norfolk 3). International journal of epidemiology. 2014;43(4):1063-72. doi: 10.1093/ije/dyt086. PubMed PMID: 23771720.
7. Khawaja AP, Chan MP, Hayat S, Broadway DC, Luben R, Garway-Heath DF, et al. The EPIC-Norfolk Eye Study: rationale, methods and a cross-sectional analysis of visual impairment in a population-based cohort. BMJ open. 2013;3(3). doi: 10.1136/bmjopen-2013-002684. PubMed PMID: 23516272; PubMed Central PMCID: PMC3612817.
8. Aulchenko YS, Heutink P, Mackay I, Bertoli-Avella AM, Pullen J, Vaessen N, et al. Linkage disequilibrium in young genetically isolated Dutch population. European journal of human genetics : EJHG. 2004;12(7):527-34. doi: 10.1038/sj.ejhg.5201188. PubMed PMID: 15054401.
9. Pardo LM, MacKay I, Oostra B, van Duijn CM, Aulchenko YS. The effect of genetic drift in a young genetically isolated population. Annals of human genetics. 2005;69(Pt 3):288-95. doi: 10.1046/j.1529-8817.2005.00162.x. PubMed PMID: 15845033.
10. Augood C, Fletcher A, Bentham G, Chakravarthy U, de Jong PT, Rahu M, et al. Methods for a population-based study of the prevalence of and risk factors for age-related maculopathy and macular degeneration in elderly European populations: the EUREYE study. Ophthalmic epidemiology. 2004;11(2):117-29. PubMed PMID: 15255027.
11. Wild PS, Zeller T, Beutel M, Blettner M, Dugi KA, Lackner KJ, et al. [The Gutenberg Health Study]. Bundesgesundheitsblatt, Gesundheitsforschung, Gesundheitsschutz. 2012;55(6-7):824-9. doi: 10.1007/s00103-012-1502-7. PubMed PMID: 22736163.

12. Holle R, Happich M, Lowel H, Wichmann HE, Group MKS. KORA--a research platform for population based health research. Gesundheitswesen. 2005;67 Suppl 1:S19-25. doi: 10.1055/s-2005-858235. PubMed PMID: 16032513.
13. Delcourt C, Diaz JL, Ponton-Sanchez A, Papoz L. Smoking and age-related macular degeneration. The POLA Study. Pathologies Oculaires Liees a l'Age. Archives of ophthalmology. 1998;116(8):1031-5. PubMed PMID: 9715683.
14. Hofman A, Darwish Murad S, van Duijn CM, Franco OH, Goedegebure A, Ikram MA, et al. The Rotterdam Study: 2014 objectives and design update. European journal of epidemiology. 2013;28(11):889-926. doi: 10.1007/s10654-013-9866-z. PubMed PMID: 24258680.
15. Wolfs RC, Borger PH, Ramrattan RS, Klaver CC, Hulsman CA, Hofman A, et al. Changing views on open-angle glaucoma: definitions and prevalences--The Rotterdam Study. Investigative ophthalmology & visual science. 2000;41(11):3309-21. PubMed PMID: 11006219.
16. Topouzis F, Coleman AL, Harris A, Jonescu-Cuypers C, Yu F, Mavroudis L, et al. Association of blood pressure status with the optic disk structure in non-glaucoma subjects: the Thessaloniki eye study. American journal of ophthalmology. 2006;142(1):60-7. doi: 10.1016/j.ajo.2006.02.055. PubMed PMID: 16815251.
17. Topouzis F, Wilson MR, Harris A, Anastasopoulos E, Yu F, Mavroudis L, et al. Prevalence of open-angle glaucoma in Greece: the Thessaloniki Eye Study. American journal of ophthalmology. 2007;144(4):511-9. doi: 10.1016/j.ajo.2007.06.029. PubMed PMID: 17893012.
18. Jacobsen BK, Eggen AE, Mathiesen EB, Wilsgaard T, Njolstad I. Cohort profile: the Tromso Study. International journal of epidemiology. 2012;41(4):961-7. doi: 10.1093/ije/dyr049. PubMed PMID: 21422063; PubMed Central PMCID: PMC3429870.
19. Bertelsen G, Erke MG, von Hanno T, Mathiesen EB, Peto T, Sjolie AK, et al. The Tromso Eye Study: study design, methodology and results on visual acuity and refractive errors. Acta ophthalmologica. 2013;91(7):635-42. doi: 10.1111/j.1755-3768.2012.02511.x. PubMed PMID: 22963377.
20. Moayyeri A, Hammond CJ, Hart DJ, Spector TD. The UK Adult Twin Registry (TwinsUK Resource). Twin research and human genetics : the official journal of the International Society for Twin Studies. 2013;16(1):144-9. doi: 10.1017/thg.2012.89. PubMed PMID: 23088889; PubMed Central PMCID: PMC3927054.
