# Supplementary material for: Increasing Prevalence of Myopia in Europe and the Impact of Education
Source: Ophthalmology. 2015 Jul;122(7):1489–97. doi: 10.1016/j.ophtha.2015.03.018 (PMC4504030; doi:10.1016/j.ophtha.2015.03.018)
Supplement: Author Study Group [file mmc3.pdf]

Members of the European Eye Epidemiology (E^3) Consortium

Niyazi Acar	Inra-University of Burgundy, Dijon, France
Eleftherios Anastosopoulos	University of Thessaloniki, Thessaloniki, Greece
Augusto Azuara-Blanco	Queen's University, Belfast, UK
Arthur Bergen	Netherlands Institute for Neurosciences-KNAW, Amsterdam, Netherlands
Geir Bertelsen	University of Tromso, Tromso, Norway
Christine Binquet	University Hospital of Dijon, Dijon, France
Alan Bird	Moorfield's Eye Hospital, London, UK
Lionel Brétillon	Inra-University of Burgundy, Dijon, France
Alain Bron	University Hospital of Dijon, Dijon, France
Gabrielle Buitendijk	Erasmus Medical Center, Rotterdam, Netherlands
Maria Luz Cachulo	AIBILI/CHUC, Coimbra, Portugal
Usha Chakravarthy	Queen's University, Belfast, UK
Michelle Chan	UCL Institute of Ophthalmology, London, UK
Petrus Chang	University of Bonn, Bonn, Germany
Annemarie Colijn	Erasmus Medical Center, Rotterdam, Netherlands
Audrey Cougnard-Grégoire	University of Bordeaux Segalen, Bordeaux, France
Catherine Creuzot-Garcher	University Hospital of Dijon, Dijon, France
José Cunha-Vaz	AIBILI/CHUC, Coimbra, Portugal
Vincent Daien	Inserm U1061, Montpellier, France
Gabor Deak	Medical University of Vienna, Vienna, Austria
Cécile Delcourt	University of Bordeaux Segalen, Bordeaux, France
Marie-Noëlle Delyfer	University of Bordeaux Segalen, Bordeaux, France
Anneke den Hollander	Radboud University, Nijmegen, Netherlands
Martha Dietzel	University of Muenster, Muenster, Germany
Maja Gran Erke	University of Tromso, Tromso, Norway
Sascha Fauser	University Eye Hospital, Cologne, Germany
Robert Finger	University of Bonn, Bonn, Germany
Astrid Fletcher	London School of Hygiene and Tropical Medicine, London, UK
Paul Foster	UCL Institute of Ophthalmology, London, UK
Panayiota Founti	University of Thessaloniki, Thessaloniki, Greece
Arno Göbel	University of Bonn, Bonn, Germany
Theo Gorgels	Netherlands Institute for Neurosciences-KNAW, Amsterdam, Netherlands
Jakob Grauslund	University of Southern Denmark, Odense, Denmark
Franz Grus	University Medical Center Mainz, Mainz, Germany
Christopher Hammond	King's College, London, UK
Catherine Helmer	University of Bordeaux Segalen, Bordeaux, France
Hans-Werner Hense	University of Muenster, Muenster, Germany
Manuel Hermann	University Eye Hospital, Cologne, Germany
René Hoehn	University Medical Center, Mainz, Germany
Ruth Hogg	Queen's University, Belfast, UK
Frank Holz	University of Bonn, Bonn, Germany
Carel Hoyng	Radboud University, Nijmegen, Netherlands
Nomdo Jansonius	Erasmus Medical Center, Rotterdam, Netherlands
Sarah Janssen	Netherlands Institute for Neurosciences-KNAW, Amsterdam, Netherlands
Anthony Khawaja	UCL Institute of Ophthalmology, London, UK
Caroline Klaver	Erasmus Medical Center, Rotterdam, Netherlands
Jean-François Korobelnik	University of Bordeaux Segalen, Bordeaux, France
Julia Lamparter	University Medical Center Mainz, Mainz, Germany
Mélanie Le Goff	University of Bordeaux Segalen, Bordeaux, France

Sergio Leal	AIBILI/CHUC, Coimbra, Portugal
Yara Lechanteur	Radboud University, Nijmegen, Netherlands
Terho Lehtimäki	Pirkanmaa Hospital District, Tampere, Finland
Andrew Lotery	University of Southampton, Southampton, UK
Irene Leung	Moorfield's Eye Hospital, London, UK
Matthias Mauschitz	University of Bonn, Bonn, Germany
Bénédicte Merle	University of Bordeaux Segalen, Bordeaux, France
Verena Meyer zu Westrup	University of Muenster, Muenster, Germany
Edoardo Midena	University of Padova, Padova, Italy
Stefania Miotto	University of Padova, Padova, Italy
Alireza Mirshahi	University Medical Center, Mainz, Germany
Sadek Mohan-Saïd	Institut de la Vision, Paris, France
Alyson Muldrew	Queen's University, Belfast, UK
Michael Mueller	Pirkanmaa Hospital District, Tampere, Finland
Sandrina Nunes	AIBILI/CHUC, Coimbra, Portugal
Konrad Oexle	Institue of Human Genetics, Munich, Germany
Tunde Peto	Moorfield's Eye Hospital, London, UK
Stefano Piermarocchi	University of Padova, Padova, Italy
Elena Prokofyeva	Inserm U1018, Paris, France
Jugnoo Rahi	UCL Institute of Ophthalmology, London, UK
Olli Raitakari	Pirkanmaa Hospital District, Tampere, Finland
Luisa Ribeiro	AIBILI/CHUC, Coimbra, Portugal
Marie-Bénédicte Rougier	University of Bordeaux Segalen, Bordeaux, France
José Sahel	Institut de la Vision, Paris, France
Aggeliki Salonikiou	University of Thessaloniki, Thessaloniki, Greece
Clarisa Sanchez	Radboud University, Nijmegen, Netherlands
Steffen Schmitz-Valckenberg	University of Bonn, Bonn, Germany
Cédric Schweitzer	University of Bordeaux Segalen, Bordeaux, France
Tatiana Segato	University of Padova, Padova, Italy
Jasmin Shehata	Medical University of Vienna, Vienna, Austria
Rufino Silva	AIBILI/CHUC, Coimbra, Portugal
Giuliana Silvestri	Queen's University, Belfast, UK
Christian Simader	Medical University of Vienna, Vienna, Austria
Eric Souied	University Hospital of Créteil, Créteil, France
Henriet Springelkamp	Erasmus Medical Center, Rotterdam, Netherlands
Robyn Tapp	Pirkanmaa Hospital District, Tampere, Finland
Fotis Topouzis	University of Thessaloniki, Thessaloniki, Greece
Virginie Verhoeven	Erasmus Medical Center, Rotterdam, Netherlands
Therese Von Hanno	University of Tromso, Tromso, Norway
Stela Vujosevic	University of Padova, Padova, Italy
Katie Williams	King's College London, London, UK
Ute Wolf-Schnurrbusch	University of Bern, Bern, Switzerland
Christian Wolfram	University Medical Center, Mainz, Germany
Jennifer Yip	UCL Institute of Ophthalmology, London, UK
Jennyfer Zerbib	University Hospital of Créteil, Créteil, France
Isabella Zwiener	University Medical Center, Mainz, Germany
